# Prevalence of Pre-existing Antibodies to CRISPR-Associated Nuclease Cas9 in the USA Population

**DOI:** 10.1016/j.omtm.2018.06.006

**Published:** 2018-06-15

**Authors:** Vijaya L. Simhadri, Joseph McGill, Shane McMahon, Junxia Wang, Haiyan Jiang, Zuben E. Sauna

**Affiliations:** 1 Hemostasis Branch, Division of Plasma Protein Therapeutics, Office of Tissues and Advanced Therapeutics, Center for Biologics Evaluation and Research, U.S. Food and Drug Administration, Silver Spring, MD 20993, USA; 2 Editas Medicine, Cambridge, MA 02141, USA

**Keywords:** CRISPR/Cas9, immunogenicity, anti-drug antibodies, pre-existing antibodies, gene editing, drug development

## Abstract

The repurposing of the CRISPR/Cas microbial adaptive immune system for gene editing has resulted in an exponential rise in new technologies and promising approaches for treating numerous human diseases. While some of the approaches being currently developed involve *ex vivo* editing by CRISPR/Cas9, many more potential applications will require *in vivo* editing. The *in vivo* use of this technology comes with challenges, one of which is the immune response to Cas9, a protein of microbial origin. Thus, the prevalence of pre-existing antibodies to Cas9 could also be a relevant parameter. There are many avenues for how CRISPR/Cas9 technologies will be applied *in vivo*, including the mode of delivery. These may be expected to invoke different immunological pathways. Nonetheless, as with all protein therapeutics, it may be desirable to monitor for anti-Cas9 antibodies during clinical development. This will require the development of robust and reliable assays. Here, we describe ELISA-based assays that are capable of detecting antibodies to Cas9 from *Staphylococcus aureus* (SaCas9) and *Streptococcs pyogenes* (SpCas9) in human sera. Furthermore, using these assays to screen for pre-existing antibodies in 200 human serum samples, we found the prevalence of anti-SaCas9 and anti-SpCas9 antibodies to be 10% and 2.5%, respectively.

## Introduction

CRISPR/Cas9-mediated genome-editing technology is not only a versatile scientific tool for addressing diverse questions in basic biology,^1^ it also holds immense promise in treating numerous human diseases.^2^ However, Cas9 proteins are derived from *Staphylococcus aureus* (SaCas9) and *Streptococcus pyogenes* (SpCas9) bacteria, which are common human pathogens, and prior exposure may result in anti-Cas9 antibodies in humans. Indeed, a recent report suggested that a high proportion of the population may have pre-existing anti-Cas9 antibodies, 79% for SaCas9 and 65% for SpCas9, based on western blotting of serum samples from 22 healthy cord blood and 12 adult donors.^3^

The presence of pre-existing antibodies to Cas9 proteins does not necessarily mean that the efficacy of Cas9-mediated gene editing will be compromised, but such knowledge may factor into risk-benefit analyses for individual patients. First, it is necessary to develop and validate a reliable bioassay to determine whether anti-Cas9 antibodies neutralize (inhibit) Cas9 activity. Second, the impact of neutralizing Cas9 antibodies needs to be assessed in the context of individual CRISPR/Cas9 regimens. It is recognized that the clinical use of Cas9 is not likely to be comparable to that of therapeutic proteins, such as replacement proteins and monoclonal antibodies. For *in vivo* viral vector-mediated gene delivery of the CRISPR/Cas9 system, Cas9 is expressed intracellularly without direct exposure to circulating pre-existing anti-Cas9 antibodies, while, for *ex vivo* cell therapy, Cas9 and guide RNA are delivered as a ribonucleoprotein complex that is present only transiently in cells prior to the infusion of the genome-edited cell product into patients.

Pre-existing antibodies to Cas9 per se may not be a significant impediment in specific clinical applications of Cas9. Nevertheless, their presence (especially at high titers) suggests that individuals likely have memory T cells and B cells that are capable of mounting an adaptive immune response to Cas9 or to cells presenting Cas9 antigenic epitopes, which could present a potential efficacy or safety concern.^4^ Bacterial proteins used in therapeutic interventions, such as pseudomonas toxin for targeted cancer therapies, have been shown to elicit strong immune responses that abolish efficacy.^5^ Therefore, assessing the immunogenicity of all CRISPR/Cas9-based therapeutic products would be desirable. Risk assessment is predicated on two questions: (1) does the therapeutic elicit anti-drug antibodies (ADAs), and (2) what, if any, are the clinical consequences of these ADAs? The first question can be addressed using a well-established standard assay development and statistical methodology for identifying positive ADA in clinical samples,^6^ which we implemented in our study. The second question needs to be addressed individually for each CRISPR/Cas9 product based on the method of Cas9 production, composition, route of administration, and target cell characteristics.

A key step in assessing immunogenicity is to establish a robust, specific, and reliable assay to detect anti-Cas9 antibodies in serum samples, either pre-existing or elicited in response to the therapeutic, in accordance with industry-authored white papers and guidance documents from the FDA and EMA.^6^, ^7^, ^8^ It is important that the assay be reliable because the results will inform the immunogenicity risk management recommended by regulatory agencies.^7^ Such an assay may even be necessary for screening potential patients prior to therapy. We report here validated ELISA-based ADA assays for the detection and quantification of anti-SaCas9 or anti-SpCas9 antibodies that can be used in both drug-naive subjects and patients treated with Cas9-based medicines. We used a tiered approach to develop screening and confirmatory assays for both anti-SaCas9 and anti-SpCas9 antibodies. Taking into consideration that normal donors may have prior exposure to Cas9 and, thus, pre-existing anti-Cas9 antibodies, we compared 2 different methods using either untreated serum samples^8^ or immune-inhibited serum samples^9^ for cut point determination in the screening assays. For both methods, statistical analyses for determining the screening cut points and assay validation were carried out using a training set of serum samples from 48 healthy donors. The prevalence of anti-SaCas9 and anti-SpCas9 antibodies in the USA population was estimated in an independent sample of sera from 200 additional donors and found to be much lower than previously suggested.^3^

## Results

### ELISA to Detect Anti-SaCas9 and Anti-SpCas9 Antibodies

**Figure 1:**
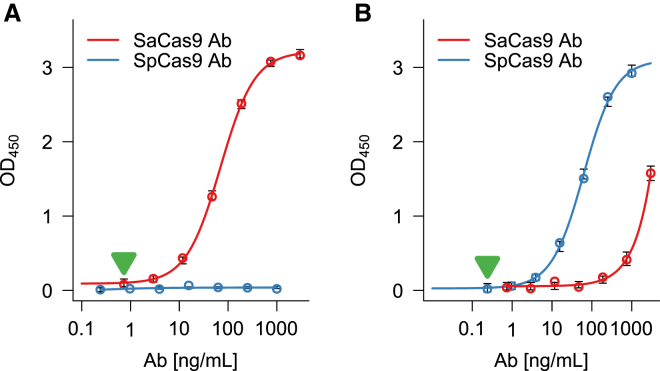
Standard Curve of Anti-SaCas9 and Anti-SpCas9 Antibody and Cross-reactivity (A and B) The plates were coated with either SaCas9 (A) or SpCas9 (B) and incubated with increasing concentrations of rabbit polyclonal anti-SaCa9 (red) or mouse monoclonal anti-SpCas9 (blue) antibodies. Data are presented as mean ± SD. The limits of quantitation for the antibodies (0.73 ng/nL for rabbit anti-SaCa9 and 0.24 ng/mL for mouse anti-SpCas9) are indicated by the green arrows.

We developed a direct format ELISA to detect anti-SaCas9 and anti-SpCas9 antibodies. We used horseradish peroxidase (HRP)-coupled protein G to detect antibodies binding to both SaCas9 and SpCas9. The assay was standardized using both rabbit polyclonal anti-SaCas9 antibody and mouse monoclonal anti-SpCas9 antibody. Figure 1A shows the concentration-response curve for varying antibody concentrations when SaCas9 was coated in the wells. The anti-SaCas9 antibody assay had a dynamic range of 0.73–750 ng/mL and a sensitivity of 0.73 ng/mL. The anti-SpCas9 antibody assay did not detect SaCa9 protein. Similarly, when SpCas9 was coated in the wells, the SpCas9-specific antibody assay had a dynamic range of 0.24–1,000 ng/mL and a sensitivity of 0.24 ng/mL. The anti-SaCas9 antibody cross-reacted with SpCas9 only at very high concentrations (>100 ng/mL) (Figure 1B).

### Assay Precision

**Figure 2:**
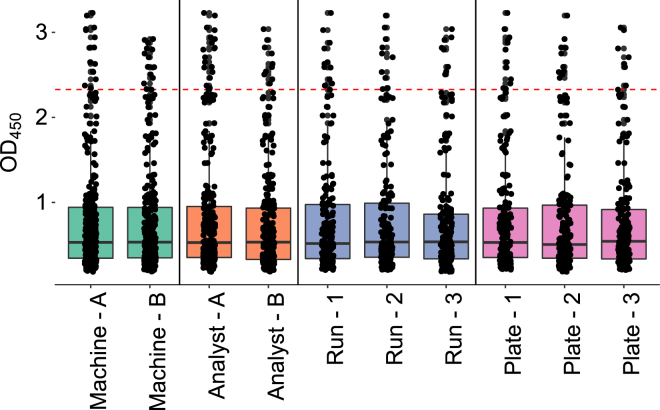
The Distribution of OD_450_ Values for SaCas9 in Serum Samples from 48 Donors in the Training Set for the Evaluation of Assay Precision The values are binned by machine (green), analyst (orange), run (blue), and plate position (pink). The box and error bar indicate the distribution of OD_450_ values for the subset of data identified. The central line shows the median OD_450_ value, the boxes indicate the range from the 25^th^ to 75^th^ percentiles, and the whiskers extend to 1.5 times the interquartile range of the data. The screening cut point for this data is shown by the red dotted line.

We carried out an experimental design that controlled for the following key assay variables: analyst, assay run, plate testing order, instrument, and sample groups tested.^8^ The experimental design used two analysts, two microplate readers, and 48 drug-naive serum samples, and it included 576 measurements (Figure 2). Additional details of the experimental design are provided in Figure S1. The results demonstrated satisfactory precision.

For each individual factor (position of sample on plate, run, analyst, or machine), the p value for the coefficient related to variance in results was above 0.05 (analyst, 0.1264; machine, 0.9248; run, 0.9281; and plate position, 0.8405), while the p value for the variance due to the sample was <0.001, leading to the conclusion that variance in the samples was due to the serum samples and not experimental influences. Additionally, Levene’s test was used to ensure that there was similar variance in the measurements based on these factors. The p value for each of these tests was well above 0.05, indicating homogeneity of variance between the different samples (analyst, 0.4197; run, 0.89028; plate position, 0.8027; and machine, 0.8441). Since the samples had equivalent means and variance, a fixed cut point was used for determining the presence of ADA.^8^

### Matrix Interference

**Figure 3:**
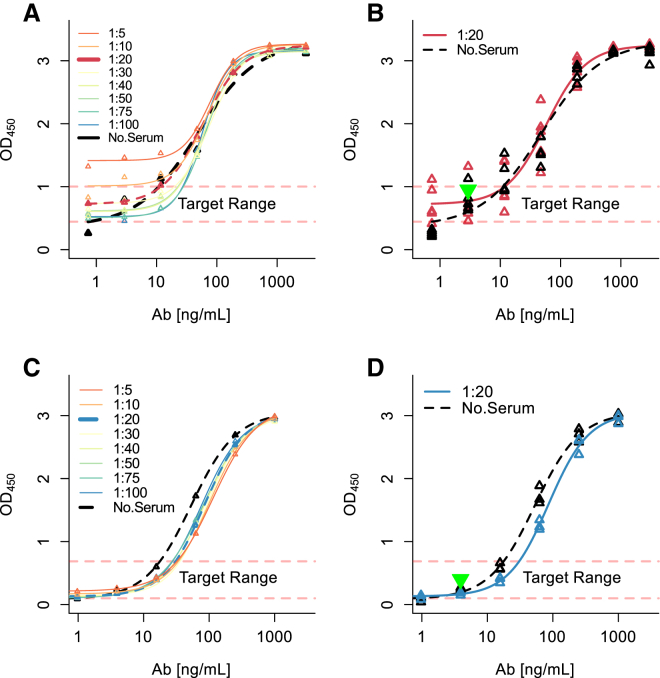
Minimum Required Dilution of Serum for the Measurement of Anti-Cas9 Antibody Levels (A–D) Five individual serum samples were spiked with increasing concentrations of anti-SaCas9 (A and B) and anti-SpCas9 (C and D) antibodies. Eight dilutions of each serum sample were used, as indicated by the colored lines. The 7-point standard curve of antibody concentration was generated by spiking the antibodies into each of 5 serum samples tested at each antibody concentration. For the detection of both anti-SaCas9 and anti-SpCas9 antibodies, a minimum serum dilution of 1:20 exhibited 80% of the dynamic range of the serum-free sample (black dashed line) (B and D). At each antibody concentration, individual OD_450_ values from serum samples are presented. Our target range for acceptable serum dilutions was the lowest 20% of the dynamic range.

While the assay described in Figure 1 had adequate sensitivity,^6^ it was conducted in assay buffer and not serum. Human antibodies in clinical or donor samples exist in a biological liquid matrix that could interfere with the assay results. Thus, we sought to identify the minimum dilution of serum that maintained at least 80% of the dynamic range of the assay. Five independent serum samples with low reactivity to SaCas9 and SpCas9 in the ELISA were used for the determination of the minimum serum dilution. Serial dilutions of each serum sample from 1:5 to 1:100 were spiked with decreasing concentrations of the SaCas9-specific antibody from 3,000 to 0.73 ng/mL or anti-SpCas antibody from 1,000 to 0.24 ng/mL in the ELISA. We found that a serum dilution of 1:20 was the minimum dilution of serum that had ≥80% of the dynamic range determined in assay buffer (Figure 3). Thus, this assay had a sensitivity of 2.93 and 3.90 ng/mL for the detection of anti-SaCas9 (Figure 3B) and anti-SpCas9 (Figure 3D) antibodies, respectively, in 1:20 diluted serum samples. The assay thus has the requisite sensitivity to detect antibodies in serum samples.^10^ In addition, the minimum required serum dilution (1:20) was well above the current recommendation (not to exceed 1:100).^6^ Taken together, these data show that the ELISA described here is suitable for detecting anti-SaCas9 and anti-SpCas9 antibodies in serum samples.

### The Screening Assay

Since only samples that are potentially positive in the screening assay are subjected to the confirmatory assay, it is critical that appropriate statistical tools and a training set with an adequate sample size is used in determining the screening cut point above which a sample is defined to be potentially positive.

First, we used the traditional method to determine the screening cut point in a drug-naive population, which consisted of a training set of 48 samples from healthy donors, assuming a false-positive rate of 5% for detecting both anti-SaCas9 and anti-SpCas9 antibodies (Figure S2). The screening cut points were 1.012 (OD_450_) and 0.874 (OD_450_) for anti-SaCas9 and anti-SpCas9 antibodies, respectively.

**Figure 4:**
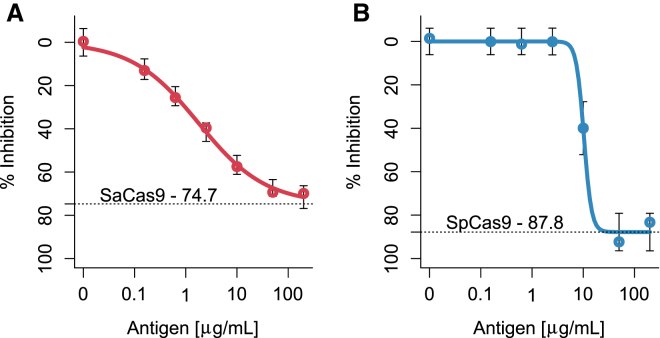
Inhibition of Anti-Cas9 Antibody Binding with Excess Antigen (A and B) The anti-SaCas9 (188 ng/mL) and anti-SpCas9 (250 ng/mL) antibodies were pre-incubated with increasing concentrations of SaCas9 (A) and SpCas9 (B) antigen. The highest concentration of antigen used (200 μg/mL) inhibited binding of anti-SaCas9 and anti-SpCas9 antibody with immobilized Cas9 by 74.7% and 87.8%, respectively.

However, since Cas9 is derived from common human pathogens, to which humans may have developed immunity from prior exposures, an assumption that the drug-naive samples are free of anti-Cas9 antibodies may result in artificially elevated screening cut points and false negatives for detecting anti-Cas9 antibodies. Therefore, we also tested an alternative immune-inhibition approach,^9^ in which excess Cas9 was added to the treatment-naive samples prior to establishing the cut point. After we determined that an excess of free Cas9 at 200 μg/mL inhibited binding of anti-SaCas9 antibodies to immobilized SaCas9 by 74.7% (Figure 4A) and anti-SpCas9 antibodies by 87.8% (Figure 4B), we preincubated the serum samples (diluted 1:20) from the 48 donors in the training set with 200 μg/mL SaCas9 or SpCas9. Using this approach, the screening cut points at the false-positive rate of 5% were found to be 0.5129 (OD_450_) and 0.6146 (OD_450_) for the positive detection of anti-SaCas9 and anti-SpCas9 antibodies, respectively (see the Materials and Methods for methodological and statistical details).

### The Confirmatory Assay

The specificity of the positive anti-Cas9 antibodies identified in the screening assay was determined using a competitive inhibition test in the confirmatory assay. All 48 serum samples in the training set at 1:20 dilution were incubated with or without an excess of free Cas9 at 200 μg/mL to determine the percent of inhibition of the specific binding between the anti-Cas9 antibodies and immobilized SaCas9. The confirmatory cut points for positive anti-SaCas9 and anti-SpCas9 antibodies were determined to be 71.61% and 73.11% inhibition. These cut points were determined using a statistical method similar to that used to obtain the screening cut points. (1) Outliers that were outside of 1.5 times the interquartile range were once again removed. (2) A value of the mean percentage inhibition plus 1.645 times the SD of the percentage inhibition was taken as the cut point for signal inhibition (see the Materials and Methods and Shankar).

### Estimating the Prevalence of Pre-existing Anti-SaCas9 and Anti-SpCas9 Antibodies

Having established the screening and confirmatory cut points using the training set, we screened serum samples from 200 donors for pre-existing anti-SaCas9 and anti-SpCas9 antibodies. The demographics of the 200 donors are shown in Table 1. We also found that 58% of the donors tested positive for Group A Streptococcus using a commercially available kit for detecting antibodies to extracellular antigens, such as streptolysin, streptokinase, hyaluronidase, DNase, and NADase. This is consistent with colonization rates of 26.2% and 9.6% previously described for *S. aureus* and *S. pyogenes* in humans.^11^

**Table 1:** Donor Population Demographics

Demographics	Number	Percentage
Race		

African American	34	17
Caucasian	153	76.5
Hispanic	10	5
Other	3	1.5

Gender		

Female	80	40
Male	120	60

Age		

19–34	76	38
35–49	58	29
50–64	50	25
65–79	16	8
Total	200	100

Table includes only the donors evaluated for Cas9 antibodies.

**Figure 5:**
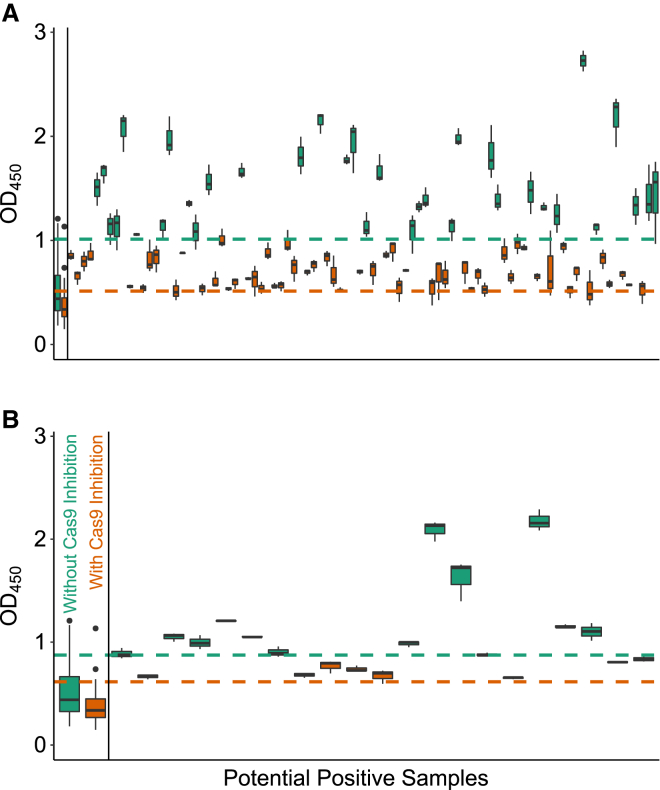
Anti-Cas9 Antibody-Positive Samples Identified in the Screening Assay The cut points defined with (orange) or without (green) inhibition by excess Cas9 competition are shown as dotted lines. (A and B) The ELISA was carried out using plates coated with SaCas9 (A) or SpCas9 (B). The bars on the extreme left indicate the values obtained using the training set as a reference point. Screening results from the 200 donors are shown for samples whose mean value for the triplicate OD_450_ reading was above the appropriate cut point. Samples identified as positive based on the cut point calculated without inhibition are shown in green. Additional positive samples identified based on the lower cut point following inhibition with excess Cas9 are shown in orange. Note that samples shown in green are also identified as positive when the lower cut point is used.

We first determined which samples were antibody-positive using cut points obtained from untreated sera (without immune-inhibition) in the training set. The results of the screening and confirmatory assays for anti-SaCas9 and anti-SpCas9 antibodies identified in 200 donors using this method are summarized in Table 2. In the screening assay, 19% and 4.5% of the donors were identified to be potentially positive for anti-SaCas9 and anti-SpCas9 antibodies, respectively (Figure 5). When the screen-positive samples were subjected to the confirmatory assay, only 5% and 1.5% of the donors tested positive for anti-SaCas9 and anti-SpCas9 antibodies, respectively.

**Table 2:** Summary of Anti-Cas9 Antibody Detection against Recombinant SaCas9 andSpCas9 Proteins by ELISA without and with Inhibition

	SaCas9				SpCas9			
	Screening		Specificity		Screening		Specificity	
	Without	With	Without	With	Without	With	Without	With
Race								

African American (n = 34)	17.6 (6)	64.7 (22)	2.9 (1)	8.8 (3)	5.8 (2)	11.8 (4)	2.9 (1)	2.9 (1)
Caucasian (n = 153)	14.3 (22)	39.2 (60)	3.9 (6)	8.5 (13)	6.5 (10)	10.5 (16)	0.7 (1)	2.0 (3)
Hispanic (n = 10)	50.0 (5)	70.0 (7)	20.0 (2)	30.0 (3)	10.0 (1)	10.0 (1)	10.0 (1)	10.0 (1)
Other (n = 3)	33.0 (1)	33.0 (1)	33.0 (1)	33.0 (1)	0.0 (0)	0.0 (0)	0.0 (0)	0.0 (0)

Gender								

Female (n = 80)	11.3 (9)	36.3 (29)	2.5 (2)	6.3 (5)	8.8 (7)	12.5 (10)	1.3 (1)	2.5 (2)
Male (n = 120)	20.8 (25)	50.8 (61)	6.7 (8)	12.5 (15)	5.0 (6)	9.2 (11)	1.7 (2)	2.5 (3)

Age								

19–34 (n = 76)	21.1 (16)	51.3 (39)	3.9 (3)	11.8 (9)	7.9 (6)	13.2 (10)	1.3 (1)	2.6 (2)
35–49 (n = 58)	12.1 (7)	41.4 (24)	5.2 (3)	6.9 (4)	6.9 (4)	8.6 (5)	1.7 (1)	1.7 (1)
50–64 (n = 50)	14.0 (7)	40.0 (20)	6.0 (3)	8.0 (4)	6.0 (3)	10.0 (5)	2.0 (1)	2.0 (1)
65–79 (n = 16)	25.0 (4)	43.8 (7)	6.3 (1)	18.8 (3)	0.0 (0)	6.3 (1)	0.0 (0)	0.0 (0)
Total (n = 200)	17.0 (34)	45.0 (90)	5.0 (10)	10.0 (20)	6.6 (13)	10.5 (21)	1.5 (3)	2.5 (5)

Proteins of interest are SaCas9 and SpCas9. Assays are screening and specificity. Inhibition (without and with) is shown as percentage (number) of responders.

Next, we carried out the same experiments but used the cut points obtained from immune-inhibited sera^9^ (Table 2). Based on the lower cut points defined by the screening assay, higher percentages of donor samples, 45% and 10.5%, were found to be positive for anti-SaCas9 (Figure 5A) and anti-SpCas9 (Figure 5B) antibodies, respectively. Following the confirmatory assay on these screen-positive serum samples, 10% and 2.5% of the donors tested positive for anti-SaCas9 and anti-SpCas9 antibodies, respectively.

Isotyping of the anti-SaCas9 antibodies showed the following immunoglobulin G (IgG) distribution: 81% IgG1, 9% IgG2, and 9% IgG3. The distribution of anti-SpCas9 IgG isotypes was 33.3% IgG1, 33.3% IgG2, and 33.3% IgG3. We did not find any anti-SaCas9 or anti-SpCas9 antibodies that were IgG4.

### Determination of Anti-SaCas9 and Anti-SpCa9 Antibody Titers in Confirmed Positive Donors

Serum anti-Cas9 antibody titers were determined in donors confirmed to be positive using the immune-inhibition method. Of the 25 serum samples that were serially titrated, the titers for SaCas9 and SpCas9 ranged from 1:64 to 1:256 (Table 3).

**Table 3:** Titers of Anti-SaCas9 and Anti-SpCas9 Antibodies in Serum Samples Testing Positive in the Confirmatory Assay

Titration	SaCas9 No. of Positive Donors	SpCas9 No. of Positive Donors
1/256	11	1
1/64	9	4

## Discussion

We describe the development of an ELISA for identifying anti-Cas9 antibodies in serum samples. The assay is fit for the intended purpose based on two important parameters, sensitivity and dynamic range. Traditionally, a sensitivity of detection of at least 250–500 ng/mL antibody has been suggested by regulatory agencies.^6^ However, recent data suggest that antibody concentrations as low as 100 ng/mL may be associated with clinical events,^10^ and, thus, the lower possible limit of detection is preferred. Using a 1:20 minimum dilution of serum samples, our assay sensitivities (2.93 and 3.90 ng/mL for anti-SaCas9 and anti-SpCas9 antibodies, respectively) well exceed the suggested criterion of 100 ng/mL. Similarly, the two assays show a broad dynamic range, i.e., over 2 logs (Figure 1), which is desirable to reliably measure a wide range of antibody concentrations. Finally, the assays are specific with minimal cross-reactivity between anti-SaCas9 and anti-SpCas9 antibodies. Although anti-SaCas9 antibody showed binding to SpCas9, this occurs only at antibody concentrations that are two logs higher than those required for binding to SaCas9.

The assay has been designed to be versatile by using HRP-coupled protein G instead of a secondary antibody. This allows the same secondary detection reagent to identify all IgG isotypes across different species and can thus be used for both pre-clinical (animal studies) and clinical studies (in humans). The assay is also precise and robust, yielding consistent results that are not dependent on the analyst, plate reader, or position of the sample on the assay plate.

The most critical aspect of designing the screening and confirmatory assays is the establishment of the cut point, which defines the threshold for a positive response. The standard methods and statistics used to assign cut points^8^ have been developed using untreated drug-naive samples, which do not generally contain anti-drug antibodies and thus the few positive samples can be excluded as statistical outliers. In the case of Cas9, it is difficult to distinguish outliers from true pre-existing anti-Cas9 antibodies caused by prior exposure to Cas9-producing pathogenic bacteria. We found that 58% of the donors used in this study tested positive for Group A *Streptococcus*.

In situations where a large proportion of the donor pool could have had prior exposure to a protein that is to be used as a therapeutic (as is the case with Cas9), an alternative approach was developed recently.^9^ This method uses an immune-inhibition approach to define the cut point in the presence of excess drug in treatment-naive samples.

In this report, we compared assay outcomes when defining the screening cut point using either the conventional or the immune-inhibition approach in a training set of 48 healthy donors. Using the cut point defined with the untreated samples in the screening assay, we found that 5% of the 200 donors were confirmed positive for anti-SaCas9 antibodies and 1.5% were confirmed positive for anti-SpCas9 antibodies. By comparison, the screening cut points are lower when using the immune-inhibition approach, where the binding of immobilized Cas9 by pre-existing anti-Cas9 antibodies in the serum samples is inhibited by excess (200 μg/mL) free SaCas9 or SpCas9. Consequently, 10% and 2.5% of the 200 donors were confirmed to be positive for anti-SaCas9 and anti-SaCas9 antibodies, respectively (Table 2), with antibody titers ranging from 1:64 to 1:256 (Table 3). The immune-inhibition approach likely provides a more accurate estimate of the prevalence of pre-existing anti-Cas9 antibodies. In addition, there was no significant difference in the prevalence of the anti-Cas9 antibodies with respect to race, gender, or age (Table 2).

The prevalence of anti-Cas9 antibodies by ELISA in our study is considerably lower than that suggested by immunoblotting in the report by Charlesworth et al.^3^ However, we note that both the assay format and number of samples evaluated differ between studies. We used an ELISA format with statistical methods that are the norm for the clinical assessment of ADA.^6^, ^8^, ^9^ There are several advantages of an ELISA over an immunoblotting assay. The ELISA format can be adapted for high-throughput screening of samples. The detection of antibody by ELISA is carried out in a solution phase with unaltered epitopes, whereas immunoblotting denatures the protein and its epitopes prior to detection. The ELISA method is quantitative and allows for the setting of statistically determined cut points, whereas immunoblotting relies on the subjective identification of bands and is dependent on the sensitivity of the detection reagents and exposure times. With respect to samples sizes, we used 48 serum samples for the training set and 200 samples for estimating the prevalence of ADA against SaCas9 and SpCas9 compared to 22 cord blood and 12 adult serum samples analyzed previously by Charlesworth et al.^3^

Current research and development programs are using viral vector-mediated delivery of Cas9 DNA^12^, ^13^, ^14^ or the delivery of CRISPR/Cas9 ribonucleoprotein complexes either directly or via nanoparticles.^15^, ^16^, ^17^ Humans exposed to Cas9 are unlikely to be immunotolerant to this protein. The closest human sequence matches that could be identified for SaCas9 and SpCas9 were pridoxyl-dependent decarboxylase domain-containing protein (9.42% sequence identity) and dystrobrevin alpha (7.96% sequence identity), respectively. While it is not possible to predict the clinical consequences of an immune response to Cas9, the immune pathways activated by different modes of delivery are predictable. For instance, the *in vivo* synthesis of Cas9 protein following delivery of Cas9-encoding DNA would be intracellular, and Cas9 peptides could potentially be processed by major histocompatibility complex (MHC) class I molecules to elicit a cytotoxic CD8+ T cell response.^18^ On the other hand, directly delivered exogenous Cas9 protein could potentially be processed by MHC class II molecules and engage with CD4+ T cells to elicit an antibody response.^18^ Thus, in addition to concerns about the effect of anti-Cas9 antibodies on efficacy, it is possible that the induction of cytotoxic T cells could be an important safety concern, as cells and tissues targeted for therapy might inadvertently become immunological targets.^19^ The phrase case by case is a common mantra in the regulatory assessment of immunogenicity risk,^20^ and it will be particularly relevant for different clinical medicines that use CRISPR/Cas9.

The usefulness of assays to measure anti-Cas9 antibodies (pre- and post-treatment) will probably depend on the specific clinical application and parameters such as *ex vivo* versus *in vivo* delivery, target organ, delivery vector, etc. More useful would be a a comprehensive immunogenicity assessment that includes the identification of T cell epitopes and characterization of T cell responses in conjuction with the identification of anti-Cas9 antibodies. T cell reponses are also useful in making histocompatibility leukocyte antigen (HLA) mediated. The HLA repertoire is one of the most diverse in the human genome, and it exhibits considerable variations among racial, ethnic, and geographic groups.^21^ Identification of T cell epitopes presented by Cas9 as well as their HLA restriction could, therefore, provide useful information about immunogenicity risk in sub-populations as well as in individuals.

In summary, we have developed a robust ELISA that is suitable for detecting pre-existing anti-Cas9 antibodies in drug-naive subjects and for monitoring the potential development of anti-Cas9 antibodies in patients to be treated with CRISPR/Cas9-based genome-editing medicines. However, as this assay was developed using the intact protein it cannot be used for identifying epitope mapping using linear peptide sequences. The clinical relevance of anti-Cas9 antibodies and the associated cellular immune responses to Cas9 on the efficacy and safety of the therapeutics are under evaluation in preclinical animal models, which will inform the product development and is necessary for bringing CRISPR/Cas9-based medicines safely to the clinic.

## Materials and Methods

### Materials

Serum samples from healthy donors were purchased from Innovative Research (Innovative Research, Novi, MI) and stored at −80°C until analysis. SaCas9 and SpCas9 proteins were custom-produced at Aldevron (Madison, WI) and determined to be >95% purity by reversed-phase high-performance liquid chromatography (RP-HPLC) and SDS-PAGE. The proteins were formulated in 10 mM HEPES (pH 7.5), 300 mM sodium chloride, 20% glycerol, and 1.0 mM Tris (2 CarboxyEthyl) Phosphine at concentrations of 6.4 and 6.2 mg/mL for SaCas9 and SpCa9, respectively.

### ELISA for Detecting Anti-SaCas9 and Anti-SpCas9 Antibodies

96-well ELISA plates (3455, Thermo Fisher Scientific, Waltham, MA) were coated with 100 μL SaCas9 or SpCas9 in 1× PBS at a concentration of 1 μg/mL. Plates were incubated at 4°C overnight and then washed three times with PBS with Tween (PBST) (0.05% Tween 20 in PBS). Plates were blocked using 200 μL/well 1% BSA (37525, Thermo Fisher Scientific, Waltham, MA) in PBST buffer, incubated for 1 hr at room temperature (RT), and then washed three times with PBST. Healthy donor human sera were diluted in blocking buffer, and 100 μL was added to each of the experimental wells in triplicate. The plates were incubated for 1 hr at RT and then washed three times with PBST. Natural protein G conjugated to HRP (ab7460, Abcam, Cambridge, MA) was diluted 1:2,000 in PBST buffer and added to the wells to detect the bound antibodies in human sera. The plates were incubated for 1 hr at RT and then washed three times with PBST. Substrate solution (5120-0075, Sera Care, Gaithersburg, MD) was added, and the optical density (OD_450 nm_) was determined after 5 min by stopping the reaction with 1% HCL-based stop solution (5150-0020, Sera Care, Gaithersburg, MD). A blank without serum was included in quadruplicate on each plate, and these values were subtracted from the values obtained in the presence of the serum samples. In the isotyping studies, HRP-coupled anti-human IgG1 (A10648, 1:2,000 dilution, Thermo Fisher Scientific, Waltham, MA), IgG2 (9080-05, 1:2,000, SouthernBiotech, Birmingham, AL), IgG3 (05-3620, 1:2,000 dilution, Thermo Fisher Scientific, Waltham, MA), and IgG4 (A10654, 1:2,000 dilution, Thermo Fisher Scientific, Waltham, MA) antibodies were used instead of natural protein G to detect the anti-SaCas9 and anti-SpCas9 antibodies.

### Streptozyme Assay

The commercially available Wampole ColorCard Streptozyme kit (WA-46050, Fisher Scientific, Waltham, MA) was used to screen for antibodies to streptococcal extracellular antigens in sera obtained from 200 healthy donors. The streptozyme assay is a hemeagglutination procedure performed on 1:100 (Isotonic Saline 0.85%) diluted sera. The qualitative test procedure was carried out per the manufacturer’s instructions. Briefly, the diluted sera (50 μL) and a drop of streptozyme test reagent were mixed uniformly for 2 min and then observed under a high-intensity lamp for agglutination and graded according to a standard chart.

### Matrix Interference and Minimum Required Dilution

The minimum required dilution was established by spiking sera with increasing concentrations of either SaCas9 antibody (polyclonal antibody from 3,000 to 0.732 ng/mL, Editas Medicine, Cambridge, MA) or SpCas9 antibody (ab191468, monoclonal 1,000 to 0.24 ng/mL, Abcam, Cambridge, MA). The serum samples were diluted to 1:5, 1:10, 1:20, 1:30, 1:50, 1:75, and 1:100.

### Titration of Anti-Cas9 Antibodies

The anti-SaCas9 and anti-SpCas9 antibodies detected in serum samples were titrated using a 4-fold dilution series. The titer reported was the highest dilution with an OD_450_ value that was greater than the average value of the blank + 2 SDs in the ELISA.

### Statistics: Assay Precision

To determine the cut point, 576 measurements were made. A balanced experimental design was used to ensure that the readings were made in triplicate for each permutation of the assay condition (analyst, plate reader, run, and position of sample on the plate). The precision of the assay was evaluated using one-way ANOVA to determine the sources of variance in the results. In addition, Levene’s test was used to evaluate the difference in variance between assay conditions. If the means and variances of each sample were equivalent, a fixed cut point was used for the cut point analysis. If differences in the mean or variance could be attributed to any of these conditions, a floating cut point was used.^8^

### Determination of Screening Cut Point

The screening cut points were determined based on two variations of the assay performed using sera from 48 donors (training set). In one cut point determination, the serum samples were untreated, while in the other, the serum samples were preincubated with excess SaCas9 or SpCas9 protein (immune-inhibited samples).^9^ Each cut point was evaluated for normality to ensure accurate coverage of values when using a parametric cut point. For determining the two cut points for detecting anti-SaCas9 and anti-SpCas9 antibodies using untreated sera, the data were log-transformed to achieve normality (Shapiro-Wilke test p value > 0.10). Additionally, outliers beyond 1.5 times the interquartile range were eliminated from the calculation of the cut points. The cut point was set to the exponentiation of the mean plus 1.645 times the SD of the transformed data, to achieve a type I error of 0.05. For the immune-inhibited training sets (i.e., sera preincubated with excess SpCas9), data transformation was not sufficient to create a normalized dataset and non-parametric statistics were used. The cut point was set to the 95^th^ percentile.

### Determination of the Confirmatory Cut Point

Each serum sample was pre-incubated with excess Cas9 protein to inhibit the binding of anti-Cas9 antibodies in the ELISA. The percentage decrease in the OD_450_ value due to pre-incubation with Cas9 was used to measure the specificity of the antibodies to Cas9. Using this measure, the method for determining the cut point was the same as above, but without log-transformation. Outliers that were outside of 1.5 times the interquartile range were once again removed. A value of the mean percentage inhibition plus 1.645 times the SD of the percentage inhibition was taken as the minimum cut point for signal inhibition to show the specificity for Cas9 while maintaining a 5% false-positive rate.

### Computation of Dosage-Response Curves

All statistical analyses were performed using R version 3.3.0^22^ and the Car package.^23^ All graphics were made with ggplot2^24^ and RColorBrewer.^25^

## Author Contributions

Z.E.S. and H.J. conceptualized the study. V.L.S. and S.M. performed experiments. Z.E.S., H.J., V.L.S., J.M., and J.W. designed experiments and analyzed data. Z.E.S., V.L.S., and J.M. wrote the manuscript. Z.E.S. obtained funding.

## Supplemental Information

Document S1. Figures S1 and S2 and Table S1

Document S2. Article plus Supplemental Information
