# Supplementary material for: Prevalence of Pre-existing Antibodies to CRISPR-Associated Nuclease Cas9 in the USA Population
Source: Mol Ther Methods Clin Dev. 2018 Jun 15;10:105–12. doi: 10.1016/j.omtm.2018.06.006 (PMC6070699; doi:10.1016/j.omtm.2018.06.006)
Supplement: Document S1. Figures S1 and S2 and Table S1 [file mmc1.pdf]

OMTM, Volume *10*

Supplemental Information

Prevalence of Pre-existing Antibodies to CRISPR-Associated Nuclease Cas9 in the USA Population

Vijaya L. Simhadri, Joseph McGill, Shane McMahon, Junxia Wang, Haiyan Jiang, and Zuben E. Sauna

Figure S1: Experimental Design

Analyst	Assay Run	Assay Plate	Validation serum samples		
			S1-S16	S17-S32	S33-S48
A1	R1	P1	x		
		P2		x	
		P3			x
	R2	P1		x	
		P2			x
		P3	x		
	R3	P1			x
		P2	x		
		P3		x	
A2	R4	P1	x		
		P2		x	
		P3			x
	R5	P1		x	
		P2			x
		P3	x		
	R6	P1			x
		P2	x		
		P3		x	

A balanced experimental design was used in examining the effects of the three main variables in assay reproducibility. By assigning each sample group (S1 −S16, S17 – S32, and S33 – S48) to every permutation of analyst (A1 and A2), assay plate (P1, P2, and P3) and assay run (R1, R2, and R3), the effects of each variable can be analyzed without worry of confounding effects due to the other variables.

Figure S2: Decision making process for setting a screening cut-point

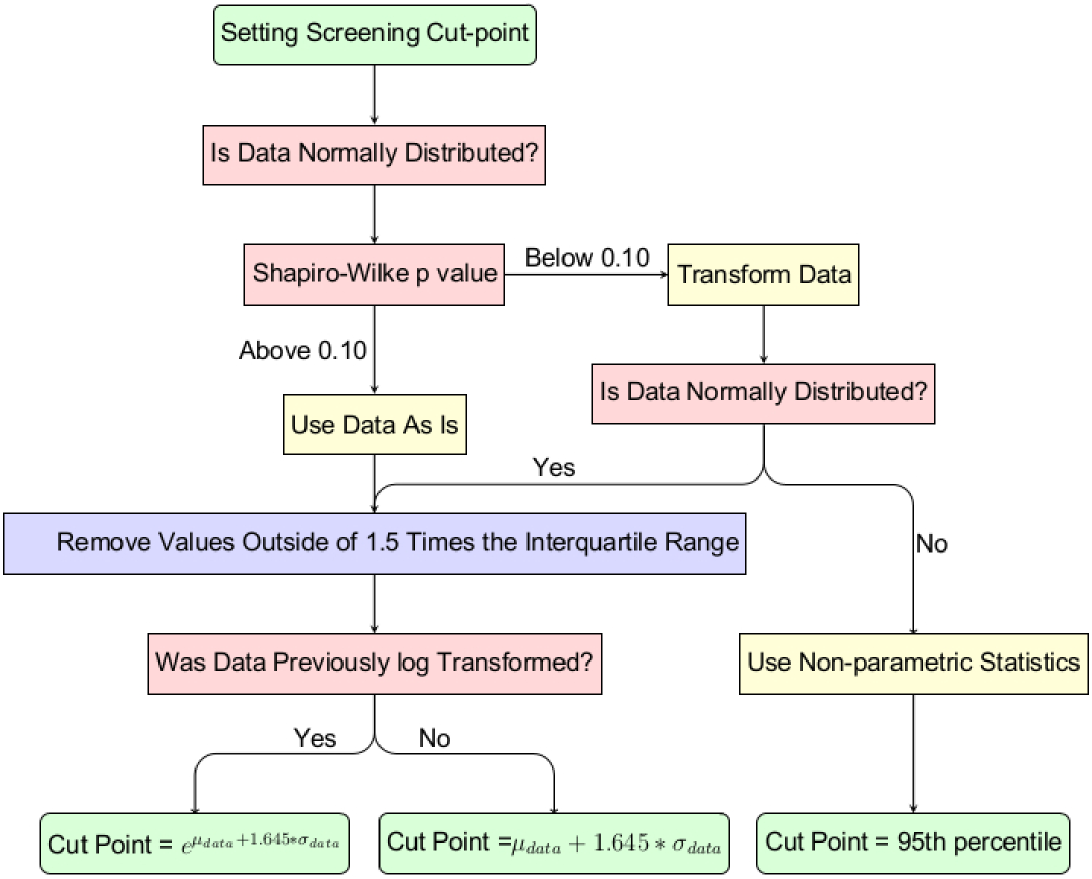

In determining the screening cut-point for the training set of donors, the data was first evaluated for normality. A log transformation was used to reshape the data and achieve normality. The cut point was then set using the mean of the transformed data plus 1.645 times the standard deviation of the transformed data. The exponentiation of this value was taken as the screening cut point. In the case of SpCas9 with the inhibition method of setting a cut point, normality could not be achieved through transformation of the data and therefore a non-parametric method was used. In all cases, the cut point was set to achieve a one-sided false positive rate of 5%.

Table S1: The titers of each positive donor are listed in the table.

SaCas9		SpCas9	
Donor	Titration	Donor	Titration
56	1/256	152	1/64
57	1/64	172	1/256
72	1/256	178	1/64
89	1/256	220	1/64
91	1/64	228	1/64
93	1/256		
121	1/64		
125	1/256		
127	1/64		
134	1/256		
146	1/256		
155	1/256		
163	1/256		
172	1/64		
181	1/64		
200	1/64		
219	1/64		
223	1/256		
241	1/64		
248	1/256		
